# Supplementary material for: Effect of Fermented Artemisia argyi on Egg Quality, Nutrition, and Flavor by Gut Bacterial Mediation
Source: Animals (Basel). 2023 Nov 28;13(23):3678. doi: 10.3390/ani13233678 (PMC10705669; doi:10.3390/ani13233678)
Supplement: Supplementary file 1 [file animals-13-03678-s001.zip › animals-2706556-supplementary.pdf]

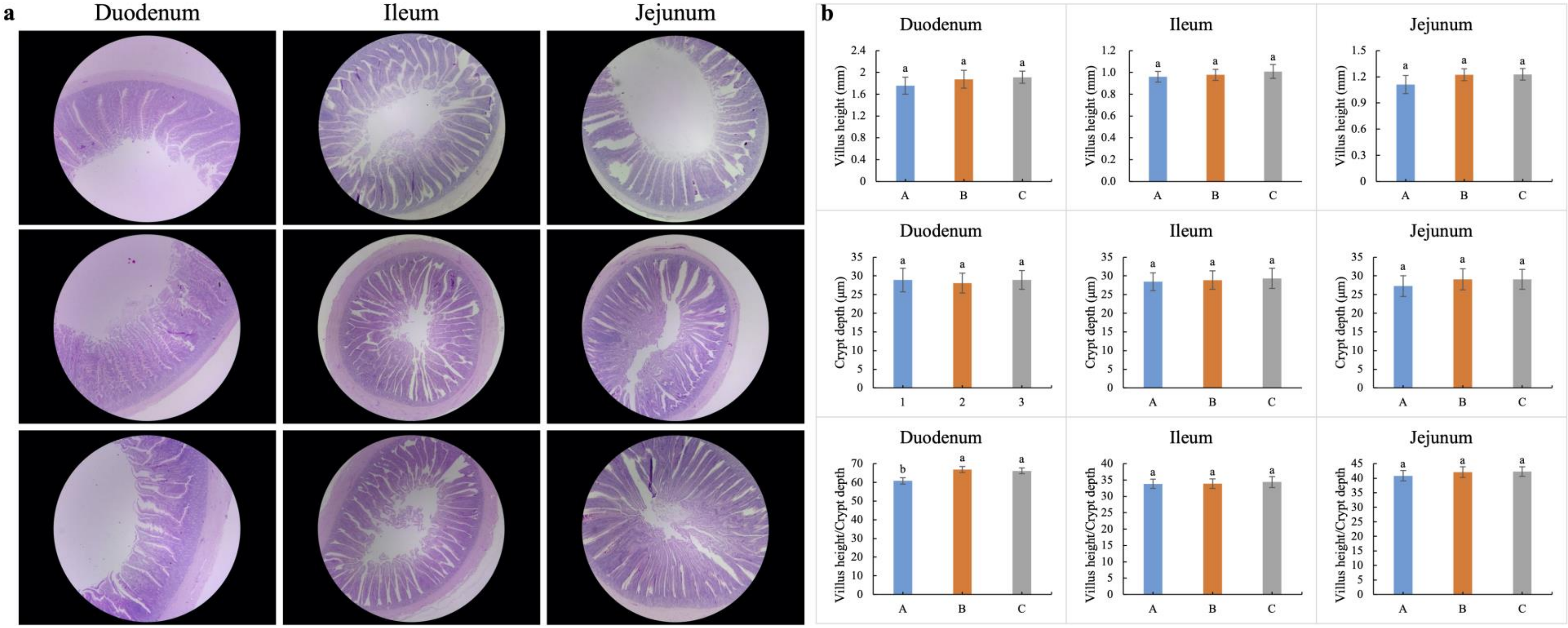

Figure S1. Effect of AAF on intestinal histology. (**a**) Representative photomicrographs (40x) of villus in the duodenum, ileum, and small intestine of the three tested groups. (**b**) Villus height, crypt depth, and villus height to crypt depth ratio of duodenum, ileum, and small intestine in the three tested groups. Different small letters manifested significant differences at $p < 0.05$ level among the three tested groups.

Table S1. The composition and nutrient levels of basal diet for hens.

Ingredient	Composition
Corn	62.73
Soybean meal	20.58
Limestone	5
Dicalcium phosphate	8.96
Soybean oil	1.27
Vitamin premix [a]	0.6
Minerals premix [b]	0.02
Choline chloride	0.1
Salt	0.3
L-Methionine	0.34
Total	100
Calculated chemical composition (%)	
ME (kcal/kg)	2658
Crude protein	17
Calcium	3.49
Total phosphorus	0.55
Lysine	0.8
Methione	0.379

[a] Vitamin premix provide the following per kilogram of diet: vitamin A, 12,500 IU; vitamin D3, 2,500 IU; vitamin E, 15 IU; vitamin K3, 2.65 mg; vitamin B1, 2 mg; vitamin B2, 6 mg; vitamin B12, 0.025 mg; nicotinic acid, 50 mg; calcium pantothenate, 12 mg; biotin, 0.0325 mg; folic acid, 1.25 mg.

[b] The mineral premix provide the following per kg of diet: iron, 80 mg; copper, 8 mg; manganese, 100 mg; zinc, 75 mg; iodine, 0.35 mg; selenium, 0.15 mg.

Table S2. The material basis of AAF.

Index	Content
Polyphenol (mg/g)	5.10±0.53
Flavonoid (mg/g)	8.22±0.61
Endotoxin (eu)	< 15
Hexachlorocyclohexane (mg/kg)	ND
Dichlorodiphenyl Trichloroethane (mg/kg)	ND
Quintozene (mg/kg)	ND
Salmonella (25 g)	ND
Staphylococcus aureus (25 g)	ND
Pb (mg/kg)	0.18 ± 0.03
Cd (mg/kg)	0.03 ± 0.01
Hg (mg/kg)	0.002 ± 0.001

Table S3. Safety Inspection of Eggs.

Item	Index	A	B	C
Microorganisms	*Salmonella* (CFU/mL)	-	-	-
	Aerobic plate count (CFU/mL)	< 100	< 100	< 100
	Moulds (CFU/mL)	< 3	< 3	< 3
	Coliforms (CFU/mL)	< 10	< 10	< 10
Aflatoxins	Aflatoxin B1 (μ/kg)	-	-	-
Heavy metal	Pb (mg/kg)	< 0.01	< 0.01	< 0.01
	Cd (mg/kg)	< 0.01	< 0.01	< 0.01

Table S4. The detected volatile components in yolks. MW was the molecular weight; RI was the retention index; RT was the retention time; DT was the migration time; RIP Rel referred to the normalized treatment.

Count	Compound	CAS#	Formula	MW	RI	Rt [sec]	Dt [RIP Rel]	Comment
1	Benzaldehyde	C100527	C7H6O	106.1	1549.8	1416.033	1.16375	
2	Acetic acid	C64197	C2H4O2	60.1	1504.4	1283.096	1.05957	monomer
3	Acetic acid	C64197	C2H4O2	60.1	1503.7	1281.169	1.151	dimer
4	1-Octen-3-ol	C3391864	C8H16O	128.2	1480.9	1219.517	1.17438	
5	Methional	C3268493	C4H8OS	104.2	1473.6	1200.251	1.10209	
6	(E)-2-Octenal	C2548870	C8H14O	126.2	1437.5	1109.7	1.34236	
7	Ethyl octanoate	C106321	C10H20O2	172.3	1432.6	1098.141	1.46355	
8	(Z)-Hex-3-enol	C928961	C6H12O	100.2	1417.0	1061.535	1.26156	
9	Nonanal	C124196	C9H18O	142.2	1401.7	1026.856	1.48269	
10	1-Hexanol	C111273	C6H14O	102.2	1368.4	955.202	1.3342	
11	Ethyl heptanoate	C106309	C9H18O2	158.2	1340.6	899.412	1.39373	
12	(E)-2-Heptenal	C18829555	C7H12O	112.2	1332.8	884.302	1.26018	monomer
13	(E)-2-Heptenal	C18829555	C7H12O	112.2	1332.2	883.14	1.66888	dimer
14	2,5-Dimethylpyrazine	C123320	C6H8N2	108.1	1332.2	883.14	1.09767	
15	1-Hydroxy-2-propanone	C116096	C3H6O2	74.1	1314.3	849.433	1.04618	
16	1-Octen-3-one	C4312996	C8H14O	126.2	1310.5	842.46	1.26823	
17	Octanal	C124130	C8H16O	128.2	1298.3	820.376	1.40338	
18	4-Methyl-1-pentanol	C626891	C6H14O	102.2	1296.9	818.052	1.64796	
19	Cyclohexanone	C108941	C6H10O	98.1	1295.4	815.372	1.16098	
20	1-Pentanol	C71410	C5H12O	88.1	1264.8	765.254	1.25585	monomer
21	1-Pentanol	C71410	C5H12O	88.1	1264.8	765.254	1.50884	dimer
22	2-Methylpyrazine	C109080	C5H6N2	94.1	1275.9	783.004	1.09378	
23	Acetoin	C513860	C4H8O2	88.1	1268.8	771.518	1.0503	

24	2-Pentylfuran	C3777693	C9H14O	138.2	1241.2	728.709	1.25981	
25	(E)-2-Hexenal	C6728263	C6H10O	98.1	1232.1	715.136	1.1847	
26	3-Methyl-1-butanol	C123513	C5H12O	88.1	1220.7	698.43	1.24795	
27	Cyclopentanone	C120923	C5H8O	84.1	1214.9	690.077	1.09773	
28	Heptanal	C111717	C7H14O	114.2	1197.8	666.062	1.33096	monomer
29	Heptanal	C111717	C7H14O	114.2	1197.0	665.018	1.70056	dimer
30	2-Heptanone	C110430	C7H14O	114.2	1192.5	658.753	1.26771	
31	Isoamyl isovalerate	C659701	C10H20O2	172.3	1299.0	821.636	1.46733	
32	1-Penten-3-ol	C616251	C5H10O	86.1	1177.1	626.385	0.94752	
33	Ethyl crotonate	C623701	C6H10O2	114.1	1177.6	627.429	1.58592	
34	1-Butanol	C71363	C4H10O	74.1	1161.6	595.062	1.18668	monomer
35	1-Butanol	C71363	C4H10O	74.1	1161.6	595.062	1.37642	dimer
36	(E)-2-Pentenal	C1576870	C5H8O	84.1	1149.2	571.047	1.10959	monomer
37	(E)-2-Pentenal	C1576870	C5H8O	84.1	1150.3	573.135	1.36456	dimer
38	p-Xylene	C106423	C8H10	106.2	1146.5	565.826	1.07797	
39	2-Butylfuran	C4466244	C8H12O	124.2	1135.7	545.988	1.20842	
40	Hexanal	C66251	C6H12O	100.2	1100.3	485.429	1.26178	monomer
41	Hexanal	C66251	C6H12O	100.2	1100.9	486.473	1.56616	dimer
42	4-Methyl-3-penten-2-one	C141797	C6H10O	98.1	1126.9	530.326	1.1175	
43	2-Methyl-1-propanol	C78831	C4H10O	74.1	1109.8	501.09	1.17482	
44	1-Propanol	C71238	C3H8O	60.1	1054.5	423.276	1.11542	monomer
45	1-Propanol	C71238	C3H8O	60.1	1056.0	425.224	1.24991	dimer
46	Thiophene	C110021	C4H4S	84.1	1029.5	393.087	1.0297	
47	1-Penten-3-one	C1629589	C5H8O	84.1	1041.8	407.695	1.07995	
48	2-Butanol	C78922	C4H10O	74.1	1041.8	407.695	1.14645	monomer
49	2-Butanol	C78922	C4H10O	74.1	1041.0	406.721	1.31789	dimer

50	Isobutyl acetate	C110190	C6H12O2	116.2	1027.0	390.166	1.23513	
51	Propyl acetate	C109604	C5H10O2	102.1	1026.1	389.192	1.48194	dimer
52	Propyl acetate	C109604	C5H10O2	102.1	1026.1	389.192	1.1568	monomer
53	Pentanal	C110623	C5H10O	86.1	998.9	359.003	1.17749	monomer
54	Pentanal	C110623	C5H10O	86.1	998.9	359.003	1.42578	dimer
55	2-Pentanone	C107879	C5H10O	86.1	998.9	359.003	1.36961	
56	Ethanol	C64175	C2H6O	46.1	950.2	321.024	1.04595	monomer
57	Ethanol	C64175	C2H6O	46.1	950.2	321.024	1.1302	dimer
58	2-Methylbutanal	C96173	C5H10O	86.1	921.0	300.573	1.17601	
59	3-Methylbutanal	C590863	C5H10O	86.1	926.7	304.468	1.41395	
60	tert-Butanol	C75650	C4H10O	74.1	928.1	305.442	1.33267	
61	2-Butanone	C78933	C4H8O	72.1	915.2	296.678	1.24991	
62	Ethyl Acetate	C141786	C4H8O2	88.1	892.8	282.07	1.34153	
63	Diethyl sulfide	C352932	C4H10S	90.2	877.2	272.332	1.21148	
64	Acrolein	C107028	C3H4O	56.1	864.3	264.541	1.0829	
65	Acetone	C67641	C3H6O	58.1	839.1	249.934	1.12281	
66	Propanal	C123386	C3H6O	58.1	806.8	232.404	1.14645	
67	Ethyl formate	C109944	C3H6O2	74.1	819.6	239.221	1.21739	
68	2-Methylpropanal	C78842	C4H8O	72.1	819.6	239.221	1.28537	
69	Dimethyl sulphide	C75183	C2H6S	62.1	795.5	226.561	0.95728	
70	Acetaldehyde	C75070	C2H4O	44.1	757.7	208.059	0.98241	
71	Diethyl acetal	C105577	C6H14O2	118.2	906.4	290.835	1.03265	
72	Myrcene	C123353	C10H16	136.2	1176.4	624.86	1.21887	
73	Dimethylamine	C124403	C2H7N	45.1	1226.4	706.662	0.84792	
74	Trimethylamine	C75503	C3H9N	59.1	1227.0	707.636	0.89521	

Table S5. Effects of AAF on plasma biochemical parameters. Different small letters indicated significant differences at $p < 0.05$ level under different treatments.

	Item	A	B	C
Liver function	ALT (U/L)	2.05 ± 0.21 ^{a}	1.57 ± 0.17 ^{b}	1.62 ± 0.18 ^{b}
	AST (U/L)	193.12 ± 19.09 ^{a}	196.22 ± 10.81 ^{a}	192.53 ± 15.22 ^{a}
	ALB (g/L)	18.00 ± 1.00 ^{a}	18.62 ± 0.75 ^{a}	17.43 ± 1.77 ^{a}
	ALP (U/L)	401.82 ± 22.54 ^{a}	410.52 ± 14.40 ^{a}	407.98 ± 12.18 ^{a}
Nitrogen metabolism	UREA (umol/L)	5.74 ± 0.67 ^{a}	5.76 ± 0.37 ^{a}	5.33 ± 0.53 ^{a}
	BUN (mmol/L)	0.48 ± 0.02 ^{a}	0.46 ± 0.03 ^{a}	0.46 ± 0.02 ^{a}
	UA (umol/L)	234.33 ± 13.23 ^{a}	241.61 ± 14.52 ^{a}	233.38 ± 14.51 ^{a}
Inflammatory factor	TNF-α (ng/L)	44.38 ± 1.82 ^{a}	42.95 ± 1.61 ^{a}	43.44 ± 1.29 ^{a}
	IL-1 (ng/L)	218.66 ± 6.70 ^{a}	221.95 ± 9.93 ^{a}	220.21 ± 7.58 ^{a}
	IL-6 (ng/L)	32.58 ± 1.54 ^{a}	32.24 ± 1.59 ^{a}	33.14 ± 1.79 ^{a}
